# Inhibiting DNA methylation activates cancer testis antigens and expression of the antigen processing and presentation machinery in colon and ovarian cancer cells

**DOI:** 10.1371/journal.pone.0179501

**Published:** 2017-06-16

**Authors:** Cornelia Siebenkäs, Katherine B. Chiappinelli, Angela A. Guzzetta, Anup Sharma, Jana Jeschke, Rajita Vatapalli, Stephen B. Baylin, Nita Ahuja

**Affiliations:** 1 Department of Surgery, The Sidney Kimmel Comprehensive Cancer Center, The Johns Hopkins University School of Medicine, Baltimore, Maryland, United States of America; 2 Department of Microbiology, Immunology, & Tropical Medicine, The George Washington University, Washington, District of Columbia, United States of America; 3 Department of Surgery, The University of Texas Southwestern Medical School, Dallas, Texas, United States of America; 4 Department of Urology, Robert H. Lurie Comprehensive Cancer Center, Feinberg School of Medicine, Northwestern University, Chicago, Illinois, United States of America; 5 Department of Oncology, The Sidney Kimmel Comprehensive Cancer Center, The Johns Hopkins University School of Medicine, Baltimore, Maryland, United States of America; The University of Hong Kong, HONG KONG

## Abstract

Innovative therapies for solid tumors are urgently needed. Recently, therapies that harness the host immune system to fight cancer cells have successfully treated a subset of patients with solid tumors. These responses have been strong and durable but observed in subsets of patients. Work from our group and others has shown that epigenetic therapy, specifically inhibiting the silencing DNA methylation mark, activates immune signaling in tumor cells and can sensitize to immune therapy in murine models. Here we show that colon and ovarian cancer cell lines exhibit lower expression of transcripts involved in antigen processing and presentation to immune cells compared to normal tissues. In addition, treatment with clinically relevant low doses of DNMT inhibitors (that remove DNA methylation) increases expression of both antigen processing and presentation and Cancer Testis Antigens in these cell lines. We confirm that treatment with DNMT inhibitors upregulates expression of the antigen processing and presentation molecules B2M, CALR, CD58, PSMB8, PSMB9 at the RNA and protein level in a wider range of colon and ovarian cancer cell lines and treatment time points than had been described previously. In addition, we show that DNMTi treatment upregulates many Cancer Testis Antigens common to both colon and ovarian cancer. This increase of both antigens and antigen presentation by epigenetic therapy may be one mechanism to sensitize patients to immune therapies.

## Introduction

Cancer causes nearly one out of four deaths in the United States; progress against this disease has been limited by the difficulty of therapeutically targeting cancer cells without affecting the surrounding normal cells. Therapies that activate the host immune system have shown tremendous promise for a wide variety of solid tumors, with patients exhibiting vigorous and durable responses. However, even in cancer subtypes such as melanoma or renal cancers that are sensitive to immune therapies, 40% or less of patients respond to immunotherapy [1]. Recent work has shown that drugs that inhibit an epigenetic modification, DNA methylation, can cause immune responses in tumor cells [2–5].

Epigenetic modifications regulate gene expression and allow for tissue-specific expression of transcripts during development and differentiation. DNA methylation acts as an epigenetic silencing mark when found in promoter regions of genes. Cancer cells often have markedly different “epigenomes” than normal cells and exhibit profound changes in DNA methylation of cytosines at CpG dinucleotides. These changes include global loss of methylation at regions such as repetitive elements that must be silenced for genome stability and gain of methylation at the promoter regions of tumor suppressor and other genes. DNA methyltransferase inhibitors (DNMTis) cause re-expression of genes that are silenced by promoter DNA methylation, reactivating tumor suppressor genes [6]. Transient exposure of multiple types of tumor cells to low doses of DNMTis promotes induction of apoptosis, reduced cell cycle activity, and decreased stem cell functions in cancer cells [7]. Clinical efficacy of DNMTis such as 5-azacytidine (5-AC) and 5-aza-2’-deoxycytidine (DAC) has led to FDA approval of these drugs for the pre-leukemic disorder myelodysplasia (MDS) [8]. Epigenetic therapy with DNMTis “boosts” immune signaling from tumors through activation of the interferon response by double-stranded RNA including hypermethylated endogenous retroviruses (ERVs) [3–5]. Treatment with the DNMTi 5-AC sensitizes mouse melanoma cells to subsequent anti-CTLA4 therapy [4], likely through activation of the interferon response and subsequent signaling to host immune cells. This epigenetic treatment upregulates interferon signaling in tumor cells and causes host immune cells to selectively target tumor cells for destruction.

DNMTis also upregulate the antigen presentation pathway in cancer cells. This pathway is crucial for the presentation of foreign antigens on the surface of most cell types via the major histocompatibility complex I (MHC I) complex. Genes upregulated after treatment with 5-AC include not only MHC class I (*B2M*, *HLA-A*, *HLA-B*, *and HLA-C*), but also immune proteasome subunits and ER transporters involved in antigen processing prior to presentation (*PSMB8*, *PSMB9*, and *TAP1*) [3]. 5-AC treatment has also been shown to upregulate the type 1 interferon response upstream of antigen presentation [9, 10]. Matei *et al*. showed that 5-AC treatment directly upregulates the JAK/STAT pathway [11]. JAK/STAT signaling can then cause upregulation of genes involved in antigen presentation. Separately, DNMTis can upregulate expression of cancer testis antigens (CTAs), which are expressed in early embryonic and germ cells, but generally silenced in mature somatic cells by promoter CpG island DNA methylation [9, 12]. CTAs often remain DNA methylated and silenced in cancer cells, but they can also lose methylation and become abnormally expressed [13, 14].

Here we show that colon and ovarian cancer cell lines downregulate genes involved in antigen presentation compared to normal tissues and confirm that treatment of colon cancer cells with clinically relevant low doses of DNMTis does in fact upregulate antigen presentation and cancer testis antigens by investigating a wider range of cell lines and treatment time points than had been described previously [3]. Treatment of cancer cells with 5-AC also leads to significant re-expression of *B2M*, *CALR*, *CD58*, *PSMB8*, and *PSMB9* at both the RNA and protein level in the colon and ovarian cancer cell lines. While this upregulation has been noted on the RNA level, we now provide in-depth data including RNA expression, DNA methylation, and protein analysis that further expands on previous work.

## Materials and methods

### Cell culture treatments

Cancer cell lines Caco-2, Colo201, Colo205, Colo320, DLD-1, HCT116, HT-29, LoVo, RKO, SK-CO-1, SNUC-1, SW48, SW480, and SW620 were acquired from the American Type Tissue Collection and grown according to the ATCC instructions. Ovarian cell lines were obtained from the laboratory of Dr. Dennis Slamon and included A2780, CAOV3, DOV13, EFO27, ES2, Hey, HEYC2, Kuramochi, OAW28, OAW42, OV167, OV2008, OV90, OVCA429, OVCA432, OVMANA, OVCAR3, OVCAR5, OVKATE, PEO14, SKOV3, TOV112D, and TykNu; these were maintained under the ATCC recommended conditions. Cells were treated with 500 nM of 5-AC (Sigma) every 24 hours for 3 consecutive days and harvested at 3, 7, 10, 14, and 21 days after the beginning of treatment.

### RNA extraction

Cells were harvested with TRIzol (Invitrogen) according to the manufacturer’s protocol with the exception of an additional ethanol washing step and a subsequent clean-up via the RNeasy Mini kit (Qiagen). Normal colon total RNA was obtained from BioChain as RNA isolated from a pool of 5 human colon donors (#R1234090-P).

### Quantitative RT-PCR

For colon cancer cell lines, 1μg of RNA was transcribed into cDNA using the SuperScriptTM III First-Strand Synthesis System for RT-PCR (Invitrogen) according to the manufacturer’s instructions. cDNA was diluted 1:10 and used for subsequent qRT-PCR with 1x Power SYBR Green PCR Master Mix (Applied Biosystems). 0.2 pmol of each primer and 5 μl cDNA in a total volume of 25 μl. All samples were measured in triplicate with 35 cycles at the optimal annealing temperature of each primer in an AB StepOnePlus Real-Time PCR System (Applied Biosystems). Resulting data was analyzed using the ΔΔCt method. Melting curve analyses were performed in order to visualize potential by-products. Only results with one melting curve peak were used for subsequent analysis.

For primary tissue specimens, we used a total of 7 samples (4 primary colon adenocarcinoma tumor samples and 3 normal colon tissues) obtained from the study approved by Institutional Review Board (IRB). The tumor specimens were macro-dissected to avoid any surrounding stromal tissue. All samples underwent RNA extraction followed by gene expression analysis as shown above. A qPCR expression level of 7 candidate genes B2M, CD58, PSMB8, PSMB9, TAPBP, CALR and TAP1 was measured in all the fresh frozen samples. We used glyceraldehyde 3-phosphate dehydrogenase (GAPDH) as a housekeeping gene to normalize gene expression.

For ovarian cancer cell lines, after total cellular RNA was extracted using the Trizol method (Life Technologies, Carlsbad, California), RNA concentration was determined using the Nanodrop machine and software (Thermo Fisher Scientific, Rockville, Maryland). 1 μg total RNA was used to generate cDNA with the QuantiTect Reverse Transcription Kit (Qiagen, Venlo, The Netherlands). Quantitative reverse transcription PCR (q-RT-PCR) of *DAZL* mRNA was performed using a TaqMan assay and the Applied Biosystems 7500 Fast real-time PCR system and software. TATA Binding Protein (TBP) was used as a reference gene. The ΔΔCT method was used to calculate relative expression levels. All qRT-PCR assays were carried out in triplicate and then repeated with new cDNA synthesis. Reverse transcriptase negative cDNA synthesis reactions were performed for at least one sample per plate.

### Flow cytometry

Cells were treated with 5-AC as described above and trypsinized at day 7. One million cells were suspended in 100 μl PBS. 2.5 μl anti-MHC I (HLA-A, B, C) (12–9983, eBioscience) or Isotope control (IgG2a, kappa, 12–4724, eBioscience) and 2.0 μl 7-Aminoactinomycin (7-AAD, Thermo Fisher Scientific) (final concentration 20 μg/ml) were added and incubated for 20 minutes in the dark. Cells were washed with ice cold PBS and resuspended in 450 μl PBS. Data was obtained with a FACSCalibur (BD Biosciences) and analyzed with FlowJo 10.0.6. Experiments were performed in biological triplicate and statistical analysis was done using the Student’s T Test. P < 0.05 was considered significant (with * p<0.05, ** p<0.01, *** p<0.001).

### DNA extraction

Cells were harvested with DNA extraction buffer (50 mM Tris, 50 mM EDTA, 2% SDS, pH 8.0), digested with 30–50 μl Proteinase K (Life Technologies), and incubated at 55°C overnight. Samples were transferred to a Qiagen MaXtract® tube and an equal amount of phenol/chloroform (pH 8.0.) was added. After centrifugation the aqueous phase was transferred into a new tube, and 650 μl of 100% EtOH, 200 μl of 7.5 M ammonium acetate (NH4Ac), and 2 μl Glycoblue™ were added to each tube. DNA was precipitated at -20°C for three days. Samples were spun down for 45 minutes and the DNA pellet was washed with 70% EtOH, air dried, and eluted in DEPC H_2_O.

### Methylation analysis (methylation-specific PCR and clonal bisulfite sequencing)

DNA was bisulfite treated with the EZ DNA methylation kit (Zymo) with slight variations from the manufacturer’s protocol. In brief, 5 μl dilution buffer was added to 2 μg of DNA in a total volume of 50 μl and incubated at 37°C for 15 min. CT Conversion reagent was prepared according to the manufacturer’s protocol and 100 μl were added to each sample and incubated in the dark at 55°C for 16 hours. 400 μl binding buffer was added and sample was cleaned up with the provided spin column (two washing steps with 200 μl, incubation with desulphonation buffer for 15 minutes, two washing steps with 200 μl and final elution in 80 μl H_2_O).

Methylation-specific PCR (MSP) was performed in 25 μl reactions with 1X Magic Buffer (0.1 M (NH4)2SO4, 0.2 M Tris (pH 8.8), 0.1M MgCl2, 1.44 M β-Mercaptoethanol), 2.5 nM dNTP, 0.6 pmol of each primer (for each sample the reaction was carried out with primers specific to unmethylated and methylated DNA), and 0.04 μl Jump-Start REDTaq with 35 PCR cycles. PCR products were visualized with 2% SB (1M Boric acid, 20 M NaOH, pH 8.0) agarose gels with GelStar Nucleic Acid Gel Stain (Lonza, Rockland, ME, USA).

### Methylation analysis (450k array)

DNA was extracted as described above and processing was performed as previously published. [3] Data analysis was performed using the R statistical computing platform and methylation array data was read using the Methylium software project. [15, 16] Sample homogeneity was ensured after examining Spearman and Pearson correlation plots and Scree plots of variance. Data was expressed as a beta value, 0 representing unmethylated and 1 representing methylated sites.

### Agilent 44K expression array

RNA was extracted as described above and RNA processing for the expression array was performed as previously published. [3] Data analysis was performed using the R statistical computing platform and the Bioconductor bioinformatics software project. [16, 17] Within array normalization was performed using Loess while between array normalization was performed using aquantile. Sample homogeneity was ensured after examining Spearman and Pearson correlation plots and Scree plots of variance. Data are presented as log_2_ fold change as compared to a baseline, untreated sample.

## Results

**Fig 1:**
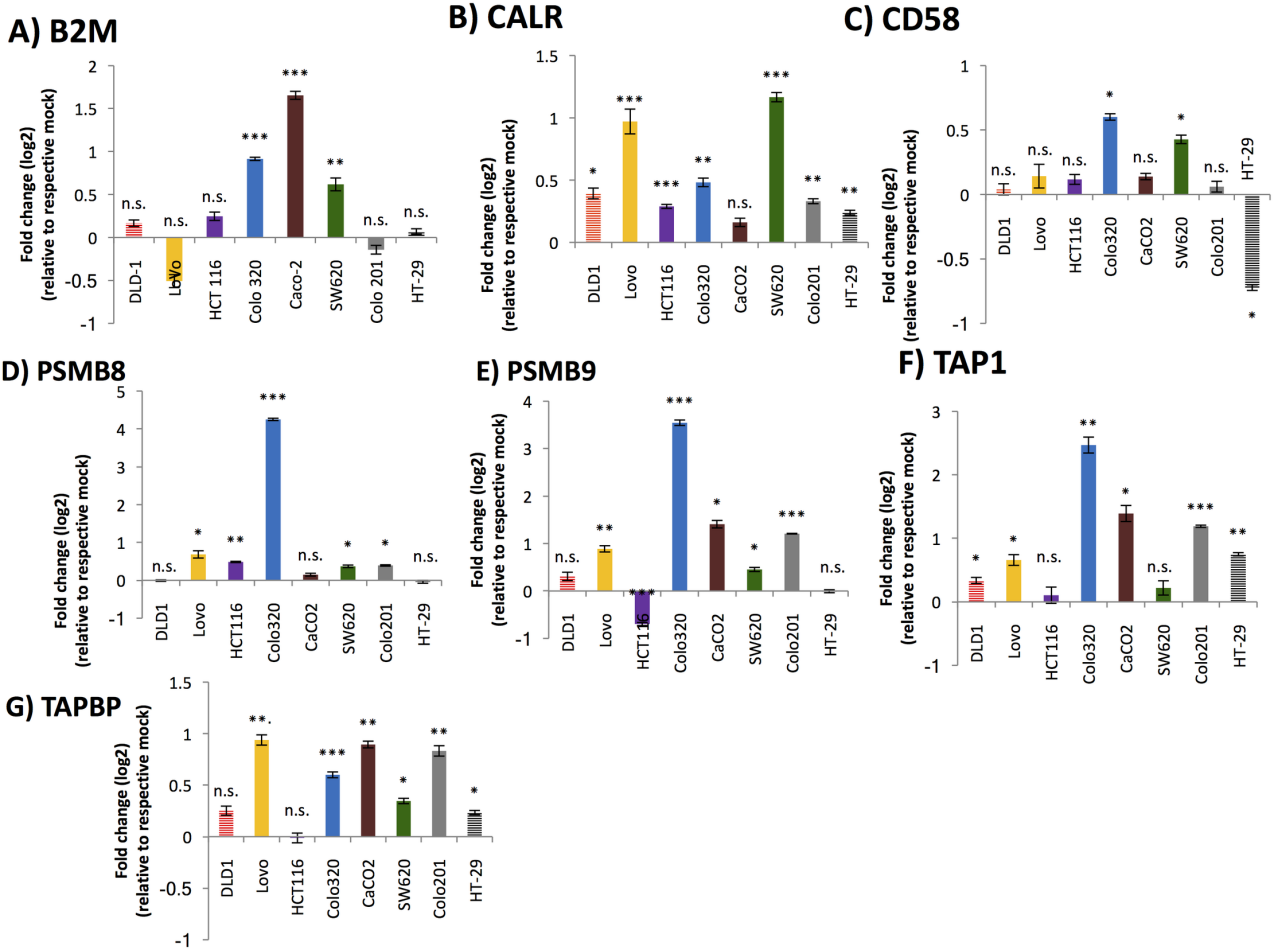
5-Azacytidine treatment leads to significant re-expression of genes involved in antigen processing and presentation. Colon cancer cell lines DLD-1, Lovo, HCT-116, Colo320, SW620, Colo201, and HT-29 were treated with 500 nM of 5-AC every 24 hours for 3 consecutive days and harvested at 10 days after the beginning of treatment. RNA was isolated and made into cDNA. qRT-PCR was performed on *B2M* (Fig 1A), *CALR* (Fig 1B), *CD58* (Fig 1C), *PSMB8* (Fig 1D), *PSMB9* (Fig 1E), *TAP1* (Fig 1F), and *TAPBP* (Fig 1G). Data is presented as log_2_ fold change over mock (untreated cells). Significance is indicated on graphs (n.s. = not significant, * = p <0.05, ** = p < 0.005, *** = p < 0.001).

**Fig 2:**
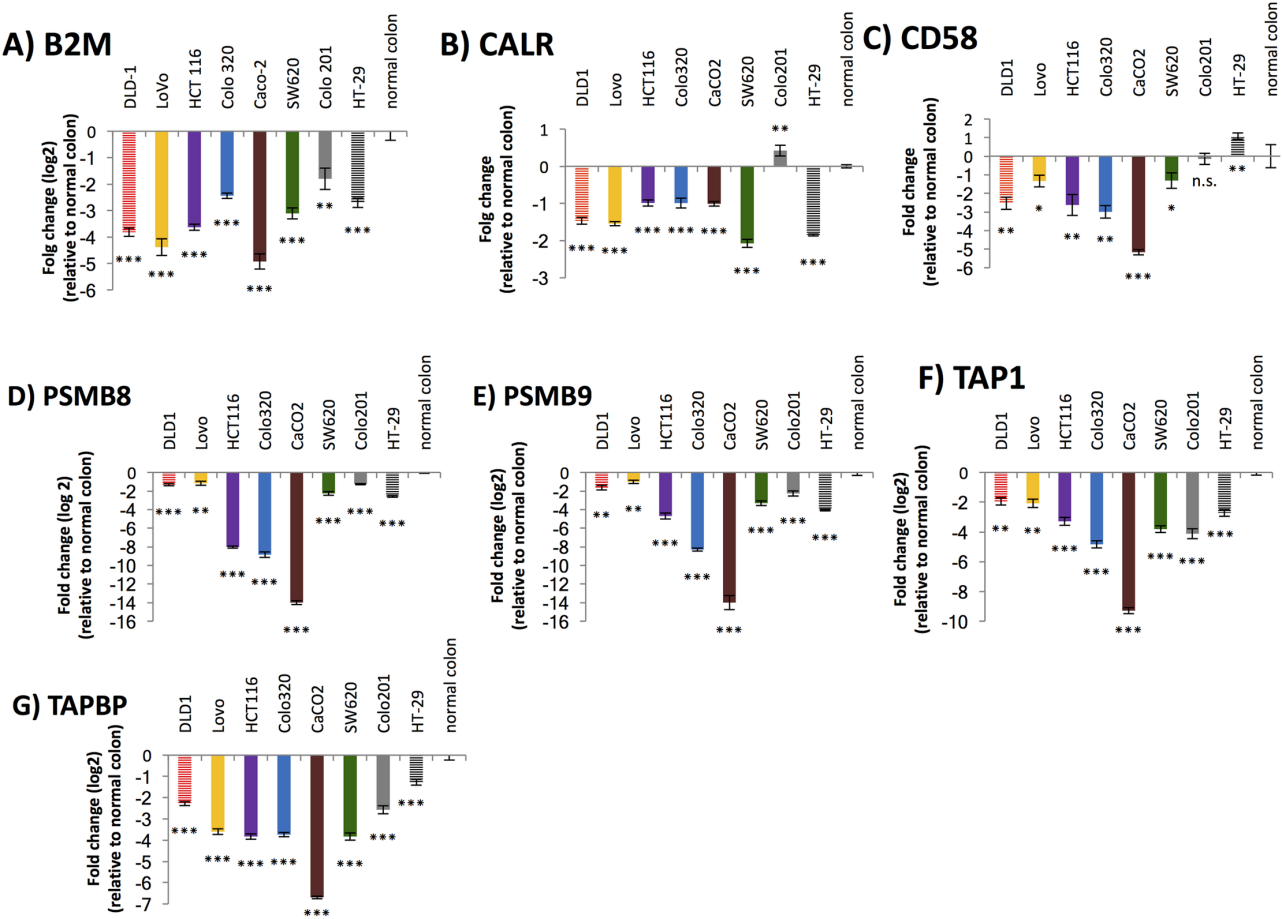
Colon cancer lines downregulate antigen processing and presentation molecules relative to normal colon. RNA was isolated from normal colon and colon cancer cell lines DLD-1, Lovo, HCT-116, Colo320, SW620, Colo201, and HT-29. cDNA was made and qRT-PCR was performed on *B2M* (Fig 2A), *CALR* (Fig 2B), *CD58* (Fig 2C), *PSMB8* (Fig 2D), *PSMB9* (Fig 2E), *TAP1* (Fig 2F), and *TAPBP* (Fig 2G). Data is presented as log_2_ fold change compared to normal colon. Significance is indicated on graphs (n.s. = not significant, * = p <0.05, ** = p < 0.005, *** = p < 0.001).

To test whether multiple antigen processing and presentation genes were increased by DNMTi treatment, we performed qRT-PCR on 8 colon cancer cell lines with and without 5-AC treatment. As previously shown [3], 5-AC upregulated antigen processing and presentation genes at the transcript level. *B2M*, which encodes the small chain of the MHC I molecule crucial for MHC I assembly, was expressed at lower levels in colon cancer cell lines relative to normal colon (Fig 1A, Fig 2A, S1A Fig) However, 3/8 colon cancer cell lines significantly upregulated *B2M* after treatment with low doses of 5-AC (Fig 1A). *CALR* encodes a protein that resides within the endoplasmic reticulum where it acts as a chaperone and facilitates the folding and association of the heavy and light chain of the MHC I complex. It is expressed at lower levels in colon cancer cell lines when compared to normal colon. When treated with 5-AC, 7/8 colon cancer lines showed significant upregulation of *CALR* (Fig 1B, Fig 2B, S1B Fig).

The protein encoded by *CD58* is not a part of the MHC I complex but is an important co-stimulatory molecule, important for the adhesion and activation of cytotoxic T cells and natural killer cells via their CD2 receptor [18]. In 7/8 tested colon cancer lines, *CD58* was downregulated when compared with normal colon. Significant upregulation of *CD58* occurred in 2/8 colon cancer lines when treated with 5-AC (Fig 1C, Fig 2C, S1C Fig). *PSMB8* and *PSMB9* encode subunits of the immunoproteasome that cleaves proteins within the cytosol into small peptides that are later presented on the cell surface via the MHC I complex. *PSMB8* was significantly downregulated in 8/8 colon cancer cell lines compared to normal colon. When the cancer cells were treated with 5-AC, 5/8 showed a significant upregulation of *PSMB8* (Fig 1D, Fig 2D, S1D Fig). Similarly, *PSMB9* was downregulated significantly in all 8 of the colon cancer cell lines we tested compared to normal colon. Upon treatment with 5-AC, 5/8 cell lines showed a significant upregulation of *PSMB9* (Fig 1E, Fig 2E, S1E Fig). *TAP1* encodes a protein that is part of a transporter that carries peptides previously cut by the proteasome from the cytosol into the endoplasmic reticulum, where the peptide is loaded upon the MHC I complex. Out of the 8 colon cancer cell lines that we investigated, all 8 showed significant downregulation of *TAP1* compared to normal colon cells. When treated with 5-AC, 6/8 of the cancer lines showed a significant upregulation of *TAP1* (Fig 1F, Fig 2F, S1F Fig). TAPBP, which mediates the interaction between MHC and TAP1 and facilitates the loading of the peptide onto the MHC I complex, showed similar results. All 8 colon cancer lines downregulated *TAPBP* compared to normal colon, but 6 of these cell lines showed a comparative upregulation when treated with 5-AC (Fig 1G, Fig 2G, S1G Fig). Several of these antigen processing and presentation genes, with B2M being the most significant, were higher on average in three normal colon samples compared to four colon cancer samples (S1H Fig).

**Fig 3:**
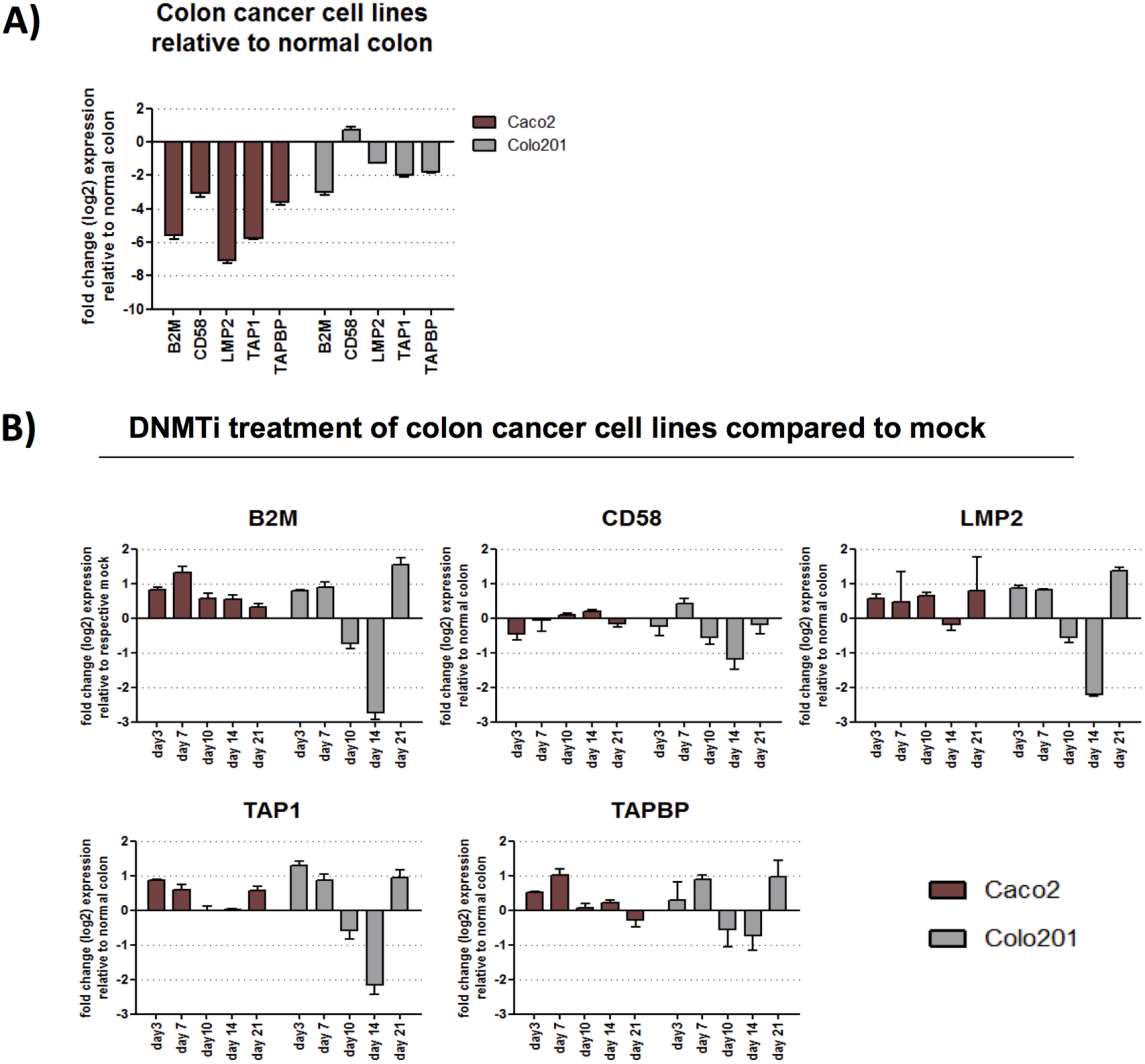
Time course of antigen processing gene expression. Colon cancer cell lines Caco2 and Colo201 were treated with 500 nM of 5-AC every 24 hours for 3 consecutive days and harvested at 3, 7, 10, 14, 21, and 28 days after the beginning of treatment. RNA was isolated and made into cDNA. qRT-PCR was performed on *B2M*, *CD58*, *LMP2*, *TAP1*, and *TAPBP*. 3A) shows expression of antigen processing genes relative to normal colon. 3B) shows expression of antigen processing genes during a time course of 5-AC treatment. Data is presented as log_2_ fold change over mock (untreated cells).

We performed a time course of 5-AC treatment investigating expression levels of 5 different antigen related genes, focusing in depth on two colon cancer cell lines. Expression was measured over a time course of 28 days, with 5-AC treatment performed during the first three days. At day three, “Mock” (untreated) colon cancer cells showed significant downregulation of the genes investigated when compared with normal colon, except for *CD58* in the Colo201 colon cancer cell line (Fig 3A). The treated cancer cells showed a significant upregulation of these genes compared to Mock on Day 3 after treatment, except for *CD58* in CaCo2 (Fig 3B). Very similar results were seen on Day 7. By Days 10 and 14, treated Colo201 cells showed a significant down regulation of all five genes that we investigated. Treated Caco2 showed significant upregulation of two of the tested genes after 10 days (*B2M*, *CD58*) with some genes remaining upregulated after 14 days (*B2M*, *CD58*, *TAPBP*) (Fig 3B). By Day 21, all genes tested but one (*CD58* in Colo201) continued to show significant down regulation in cancer cell lines compared to normal colon. In the treated group, CaCo2 cells showed significant upregulation of *B2M*, *TAP1* and *TAPBP*. Colo201 cells showed significant upregulation of *B2M*, *LMP2*, *TAP1* and *TAPBP*. 23 ovarian cancer cell lines were also investigated [3] and demonstrated upregulation of antigen processing and presentation genes (four representative cell lines are presented in S2 Fig).

**Fig 4:**
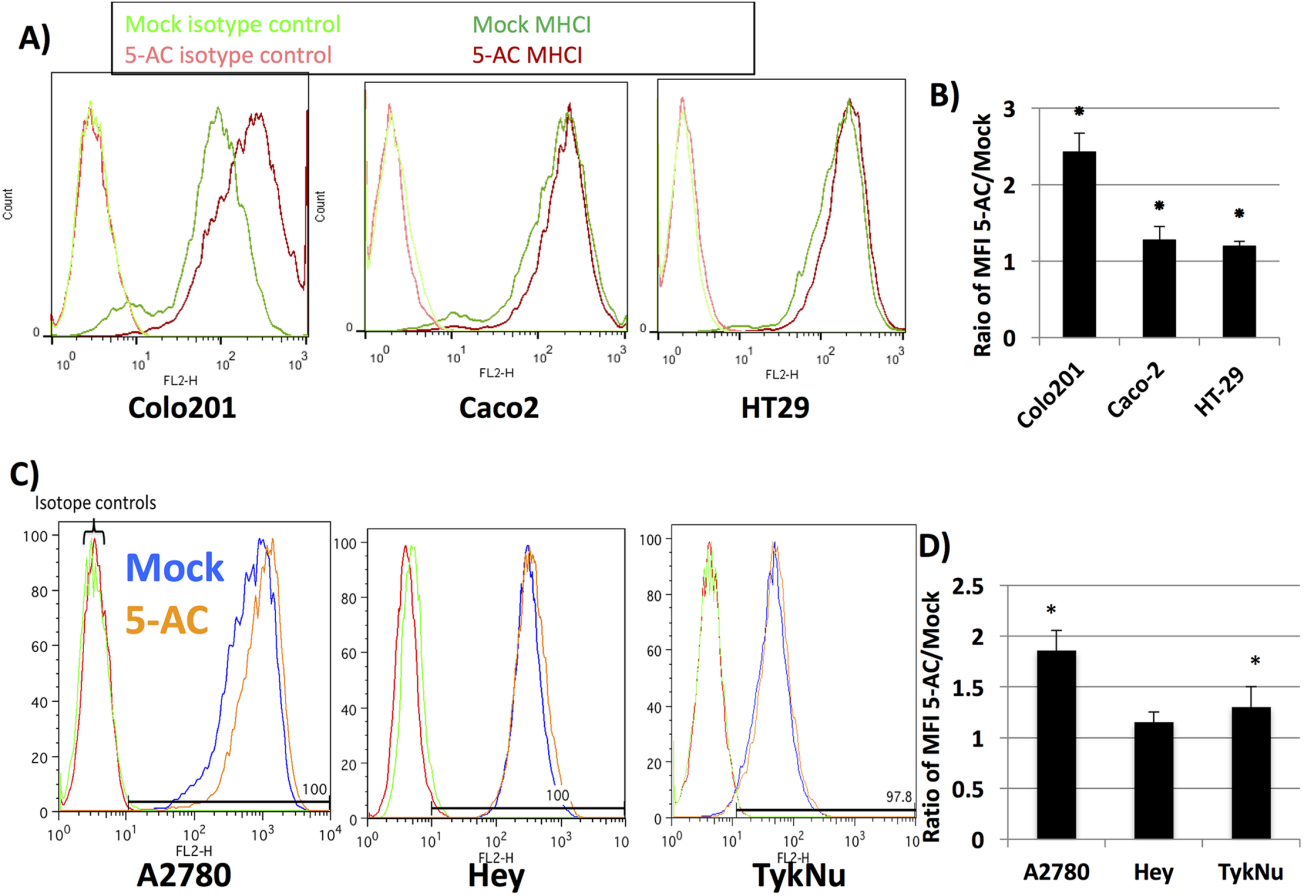
AZA upregulates the MHC I antigen presenting complex on the surface of colon and ovarian cancer cells. Colon (Fig 4A and 4B) and ovarian (Fig 4C and 4D) cancer cells were treated with 500 nM 5-AC every day for three days and trypsinized 7 days after beginning treatment. Cells were stained with anti-MHC I and 7-AAD; representative plots of MHC I staining are shown for colon (Fig 4A) and ovarian (Fig 4C) cancer cells. Plots of MFI (median fluorescence intensity) for colon (Fig 4B) and ovarian (Fig 4D) cancer cells are shown as an average of three biological replicates. * = p <0.05.

Having confirmed antigen presenting complex upregulation at the transcript level, we then assessed whether antigen presenting complex proteins were increased by 5-AC at the cell surface of the colon and ovarian cancer cells. Fig 4A shows that MHC I exhibited the greatest upregulation compared to Mock in the Colo201 line. MHC I expression in Caco2 and HT29 did not show much difference between Mock and 5-AC treated cells. In all three cell lines tested, the median fluorescence intensity suggests that the amount of MHC I increased in 5-AC treated cells when compared to the Mock treated cells (Fig 4B). In addition to colon cancer, we measured MHC I surface expression in three ovarian cancer cell lines, and one of three showed upregulation of this expression by 5-AC. Fig 4 shows this upregulation in the A2780, Hey, and TykNu ovarian cancer cell lines (FACS Fig 4C, MFI Fig 4D).

**Fig 5:**
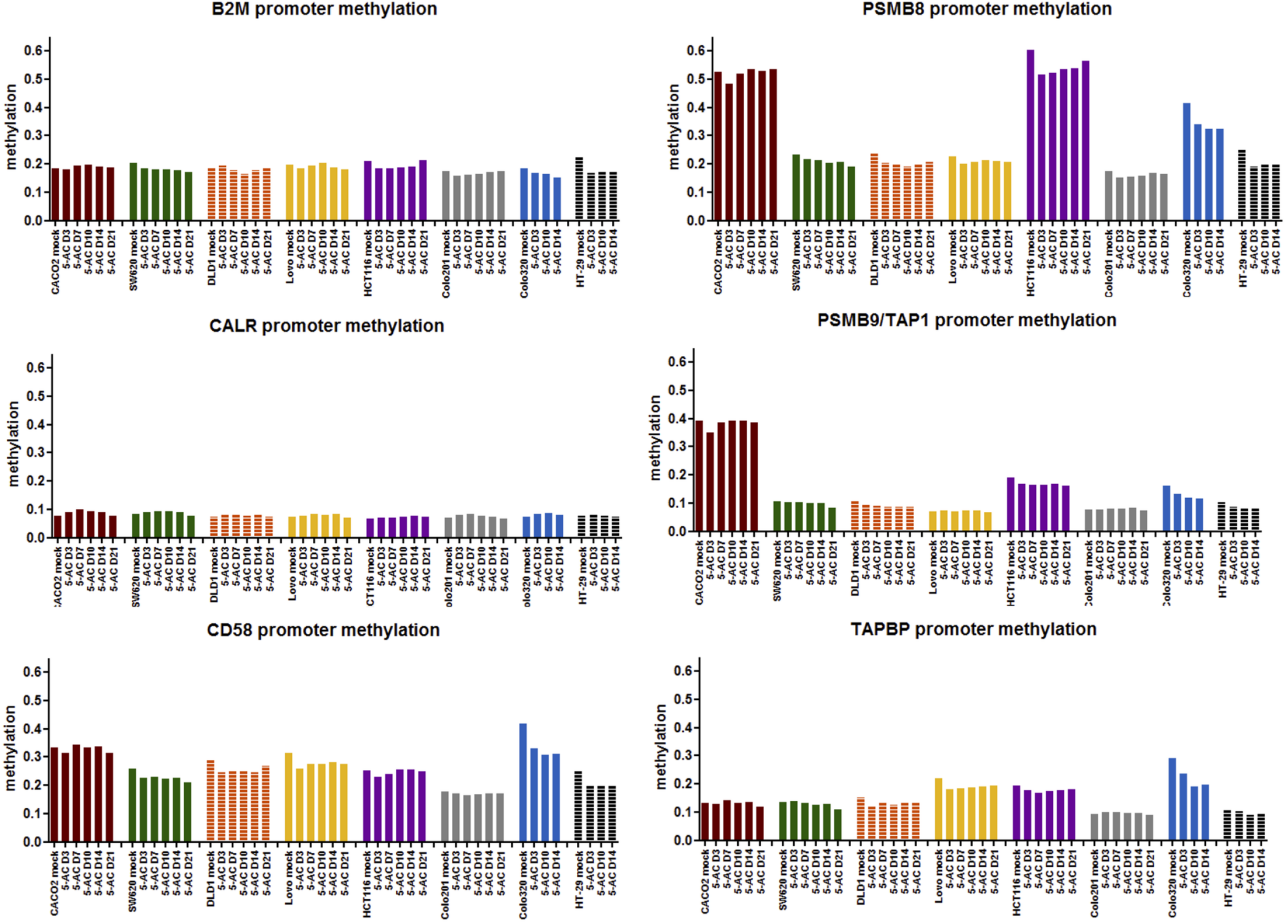
Methylation analysis at the promoter regions of antigen processing and presentation genes after 5-AC treatment. Colon cancer cell lines Caco2, SW620, DLD-1, Lovo, HCT116, Colo201, Colo320, and HT-29 were treated with 500 nM 5-AC every day for three days. DNA was extracted 3, 7, 10, 14, and 21 days after beginning treatment. DNA was analyzed using the Infinium 450k methylation array. Results are shown as percentage of methylation at the promoter region of each of the following genes: *B2M*, *PSMB8*, *CALR*, *PSMB9/TAP1* (shared promoter), *CD58*, and *TAPBP*.

While 5-AC increased the expression levels of antigen processing and presentation genes, this upregulation was not due to changes in promoter methylation. Infinium 450k methylation array analysis of *B2M*, *CALR*, *CD58*, *PSMB8*, *PSBM9/TAP1*, and *TAPBP* showed low levels of methylation initially that were unchanged by 5-AC treatment (Fig 5). Bisulfite sequencing of the *B2M* gene in Colo320, Colo201, CaCo2 and RKO showed an absence of any DNA methylation (data not shown). Methylation-specific PCR analysis of the *PSMB9* gene in the eight colon cancer cell lines investigated showed that it was unmethylated both in the untreated and 5-AC treated cell lines (S3A Fig). Methylation-specific PCR analysis of the *TAPBP* gene in the eight colon cancer cell lines investigated showed that it was partially methylated but unchanged by 5-AC treatment (S3B Fig). We hypothesize that activation of these antigen processing and presentation genes is due to upstream activation of the Type I interferon response by DNMT inhibitors. We and others [3–5, 9] have previously shown that DNMT inhibitors cause a type I interferon response, in the cell lines we have investigated in this study. *B2M*, *PSMB8*, *PSBM9/TAP1*, and *TAPBP* have all been characterized as inducible by type I interferon [19].

**Fig 6:**
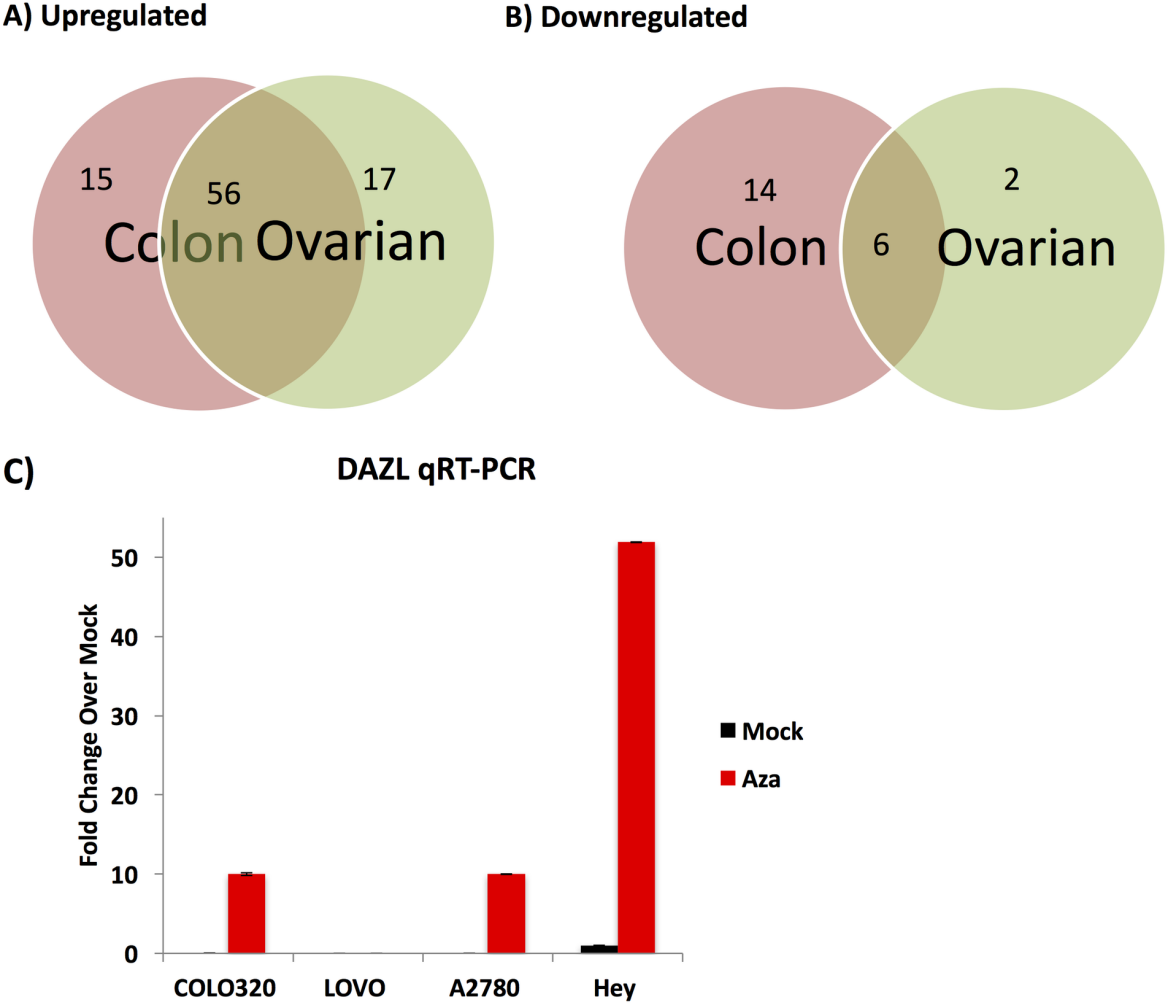
Cancer testis antigens are upregulated by 5-AC in colon and ovarian cancer cells. Colon and ovarian cancer cell lines Caco-2, Colo201, Colo205, Colo320, DLD-1, HCT116, HT-29, LoVo, RKO, SK-CO-1, SNUC-1, SW48, SW480, SW620, A2780, CAOV3, DOV13, EFO27, ES2, Hey, HEYC2, Kuramochi, OAW28, OAW42, OV167, OV2008, OV90, OVCA429, OVCA432, OVMANA, OVCAR3, OVCAR5, OVKATE, PEO14, SKOV3, TOV112D, and TykNu were treated with 500 nM of 5-AC every 24 hours for 3 consecutive days and harvested 10 days after the beginning of treatment. RNA was isolated and run on the Agilent gene expression array. Upregulated (Fig 6A) and downregulated (Fig 6B) Cancer Testis Antigens are shown 10 days after beginning 5-AC treatment. RNA was made into cDNA and qRT-PCR performed for the CTA *DAZL* in COLO320, LOVO, A2780, and Hey cell lines (Fig 6C).

Lastly, we confirmed previous results [3] showing an upregulation of cancer testis antigens (CTAs) by GSEA (Fig 6). Of 80 CTAs queried, between 71–73 were upregulated in colon and ovarian cancer cell lines, with more than half (56) in common between these two cancer types (Fig 6A). In contrast, between 8–20 CTAs were downregulated in cell lines of each cancer type, with only 6 in common (Fig 6B). At least 5 and at most 34 CTAs were significantly increased in each of 14 colon cancer and 23 ovarian cancer cell lines. We confirmed the expression array data showing upregulation of the CTA *DAZL* [20]. *DAZL* expression was increased by 5-AC treatment in one of two colon cancer lines and two of three ovarian cancer lines tested (Fig 6C). The TykNu and LOVO cell lines did not have detectable *DAZL* by qRT-PCR (data not shown). *DAZL* was especially upregulated in the Hey cell line, where it was initially methylated but became demethylated by 5-AC (Fig 6C, S4 Fig).

## Discussion

Our data builds on previous evidence that treatment of heavily methylated cancer cells with DNMTis results in an upregulation of antigen presentation genes, both at the level of the antigen presenting complex and the cancer testis antigens themselves [3]. In addition to the MHC I complex–T Cell Receptor interaction, the presenting cell and the cytotoxic T cell are also linked by the CD 58 (ligand)–CD2 (receptor) connection, which is required for adhesion and activation of T cells [21]. Although the cancer cell lines were only treated for three consecutive days and received no further treatment of 5-AC afterwards, upregulation of certain genes was still observed up to three weeks after 5-AC treatment. This ‘memory effect’ has already been shown in hematological and epithelial tumor cells by [7] and [5] and demonstrates the clinical potential of DNMT inhibition: it might be sufficient to administer this drug for only a short time to achieve a durable effect on the tumor.

MHC Class I antigens play a major role in recognition of infected or transformed cells by cytotoxic T cells that lyse those cells upon contact with their T cell receptor. This makes the MHC I molecule a frequent target of silencing in various malignancies. Components of the antigen processing machinery, which all work together to load peptides onto nascent MHC I chains, are also often downregulated [22–25]. *B2M* especially is a frequent target of mutations in cancer. In general, colorectal cancers that have downregulated MHC I expression exhibit an altered composition and intensity of infiltrating leukocytes, supporting the hypothesis that these cancers try to evade the immune system [26, 27]. 5-AC has been shown to lead to a restoration of the antigen-specific T cell response in melanoma cells due to the re-expression of the antigen processing pathway [28]. As it is crucial for the cell to achieve a balance of MHC I down regulation–a high expression leads to the recognition by T cells, but low expression attracts natural killer cells [29]–epigenetic silencing could be an efficient way for cancer cells to evade immune cells.

Upon inflammation, cells are stimulated with interferon gamma from infiltrating immune cells, leading to a change of constitutive catalytic proteasome subunits b1, b2, and b5 to the inducible subunits PSMB9 (b1i), MECL-1 (b2i), and PSMB8 (b5i) during proteasome neosynthesis. This results in the formation of the immunoproteasome which has altered substrate binding pockets [30]. The efficiency of the immunoproteasome to create peptides with a suitable length for the MHC I complex for antigen presentation is higher than the regular proteasome. This results in better presentation of antigens at the surface of cells that are in close proximity to inflammation [31]. As inflammation is a favorable environment for the initiation, promotion and invasion of cancer cells, this indicates that cancer cells exposed to inflammation have all the more reason to down-regulate the antigen processing machinery to counteract the upregulation by inflammation [32].

We propose utilizing epigenetic therapies to increase expression of both cancer testis antigens themselves as well as the antigen processing and presentation machinery. Since CTAs can be recognized by the host immune system, they represent good candidates for immune therapy, including vaccines. There is thus great potential for DNMT inhibitor treatment to upregulate CTAs on tumors, facilitating targeting by the host immune system [33–37]. For example, in a recent Phase I clinical trial in ovarian cancer, Odunsi *et al*. added DNMTi to NY-ESO-1 vaccine combined with doxorubicin chemotherapy. They observed DNA hypomethylation at the NY-ESO-1 promoter. NY-ESO-1 was upregulated and increased serum antibodies to NY-ESO-1 were detected, most importantly in two-thirds of the patients who previously were sero-negative for NY-ESO-1 antibodies. They observed specific T cell responses against NY-ESO-1 and stable disease or partial clinical response in 6/10 patients [36]. Thus DNMTi treatment may increase tumor immunogenicity at the level of the interferon response, antigen processing and presentation, and cancer testis antigen expression, reducing immune evasion and potentially synergizing with therapies that activate the host immune system.

## Supporting information

S1 Fig 5-Azacytidine treatment leads to significant re-expression of genes involved in antigen processing and presentation (array data). Colon cancer cell lines Caco-2, Colo201, Colo205, Colo320, DLD-1, HCT116, HT-29, LoVo, RKO, SK-CO-1, SNUC-1, SW48, SW480, and SW620 were treated with 500 nM of 5-AC every 24 hours for 3 consecutive days and harvested at 3, 7, 10, 14, 21, and 28 days after the beginning of treatment. RNA was isolated and made into cDNA. Agilent expression array was performed on *B2M* (Fig A), *CALR* (Fig B), *CD58* (Fig C), *PSMB8* (Fig D), *PSMB9* (Fig E), *TAP1* (Fig F), and *TAPBP* (Fig G). Data is presented as log_2_ fold change over mock (untreated cells). 4 tumors and 3 normal colon samples were macro-dissected and RNA was isolated, followed by q-RT-PCR analysis (Fig H).
(TIFF)

S2 Fig 5-Azacytidine treatment leads to significant re-expression of genes involved in antigen processing and presentation in ovarian cancer cell lines (array data). Ovarian cancer cell lines A2780, Hey, Kuramochi, and TykNu were treated with 500 nM of 5-AC every 24 hours for 3 consecutive days and harvested at 10 days after the beginning of treatment. RNA was isolated and made into cDNA. Agilent expression array was performed; data is presented for *B2M*, *CALR*, *CD58*, *PSMB8*, *PSMB9*, *TAP1*, and *TAPBP*. Data is presented as log_2_ fold change over mock (untreated cells).
(TIFF)

S3 Fig Methylation specific PCR analysis of *PSMB9* and *TAPBP* after 5-AC treatment. **Colon cancer cell lines DLD1, Lovo, HCT116, Colo320, Caco2, SW620, Colo201, and RKO were treated with 500 nM 5-AC for three days, treating every day.** DNA was isolated 7 days after beginning treatment and bisulfite treated. Methylation-specific PCR was performed on *PSMB9* (Fig A) and *TAPBP* (Fig B). “m” indicates mock sample, “a” indicates 5-AC treated sample. DKO = unmethylated control, IVD = completely methylated control, H2O = water (no template) control. Arrows to right indicate PCR bands (U = unmethylated, M = unmethylated, X = nonspecific band).
(TIFF)

S4 Fig 5-AC reduces DNA methylation at the *DAZL* promoter in the Hey cell line. Ovarian cancer cell lines were treated with 500 nM of 5-AC every 24 hours for 3 consecutive days and harvested at 3 and 10 days after the beginning of treatment. DNA was extracted and analyzed using the Infinium 450k methylation array. Results are shown as beta value (percentage methylation) at probes along the promoter region of *DAZL* (probes shown on x-axis). Blue lines indicate mock samples and red lines indicate 5-AC treated samples.
(TIFF)
