# Correction: Inhibiting DNA methylation activates cancer testis antigens and expression of the antigen processing and presentation machinery in colon and ovarian cancer cells

**DOI:** 10.1371/journal.pone.0243944

**Published:** 2020-12-10

**Authors:** Cornelia Siebenkäs, Katherine B. Chiappinelli, Angela A. Guzzetta, Anup Sharma, Jana Jeschke, Rajita Vatapalli, Stephen B. Baylin, Nita Ahuja

The raw data underlying the findings of this study [1] are missing from the list of Supporting Information. The authors have provided the data as Supporting Information file S1 Data. With this correction to [1], all relevant data are now provided.

The following information is missing from the Funding statement: This study was funded by grants to NA from Astex Inc. (GRANT # 120039) and the Van Andel Research Institute–Stand Up To Cancer Epigenetics Dream Team. Stand Up To Cancer is a program of the Entertainment Industry Foundation, administered by AACR.

There is information missing from the Competing Interests statement. The correct Competing Interests statement is as follows: The authors of this manuscript have read the journal’s policies and have the following competing interests to declare: NA was the recipient of grants from Astex Inc. and the Van Andel Research Institute. Additionally, NA has served as consultant to Johnson & Johnson, an advisor to Celgene, and a member of the Scientific Advisory Council to the No Stomach for Cancer Foundation. There are no patents or marketed products to declare. This does not affect our adheres to PLOS ONE policies on sharing data and materials.

## Supporting information

S1 Data (ZIP)
