# Supplementary figures and images for: Inhibiting DNA methylation activates cancer testis antigens and expression of the antigen processing and presentation machinery in colon and ovarian cancer cells
Source: PLoS One. 2017 Jun 16;12(6):e0179501. doi: 10.1371/journal.pone.0179501 (PMC5473589; doi:10.1371/journal.pone.0179501)

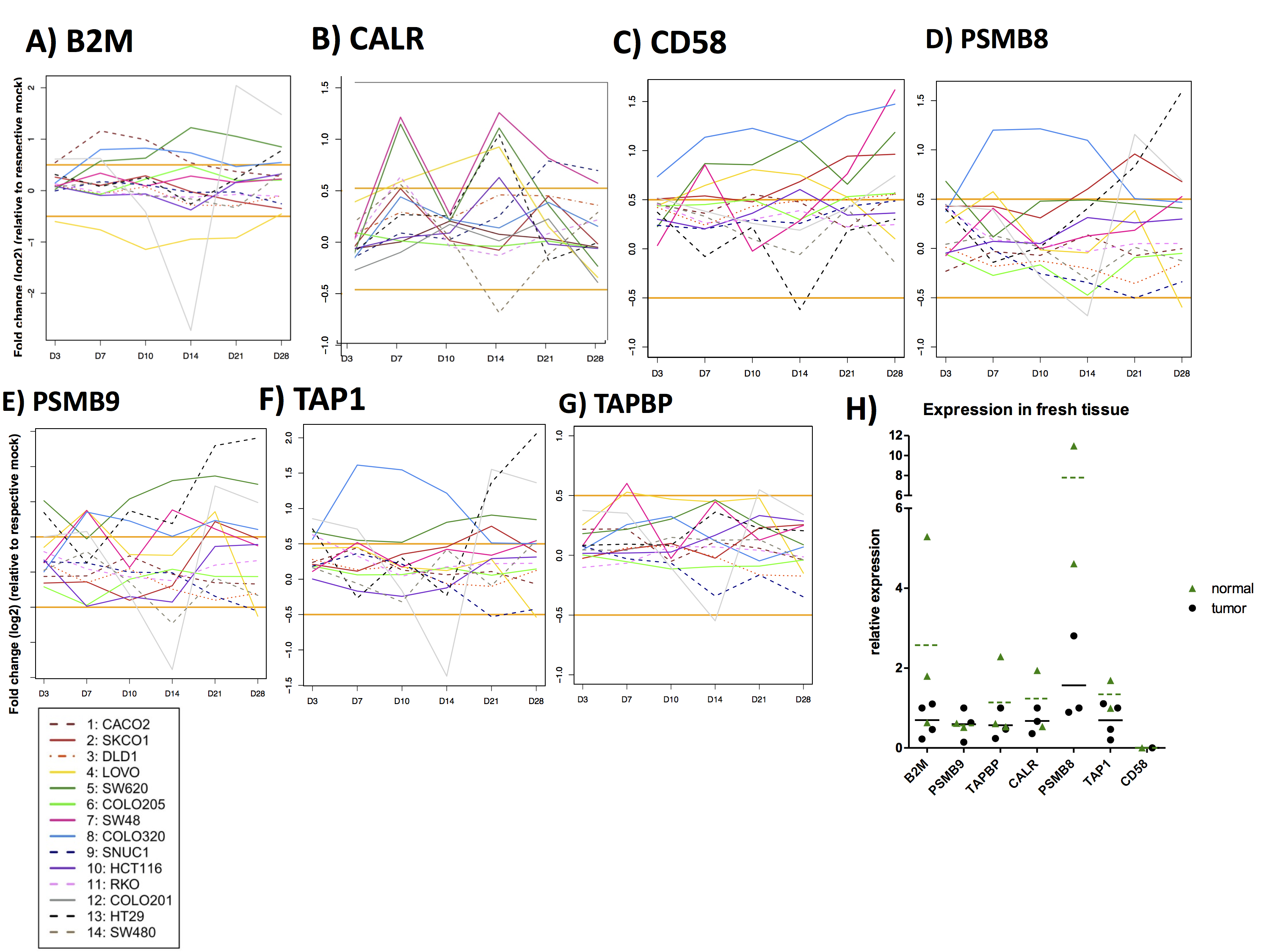

Supplement: S1 Fig — Colon cancer cell lines Caco-2, Colo201, Colo205, Colo320, DLD-1, HCT116, HT-29, LoVo, RKO, SK-CO-1, SNUC-1, SW48, SW480, and SW620 were treated with 500 nM of 5-AC every 24 hours for 3 consecutive days and harvested at 3, 7, 10, 14, 21, and 28 days after the beginning of treatment. RNA was isolated and made into cDNA. Agilent expression array was performed on B2M (Fig A), CALR (Fig B), CD58 (Fig C), PSMB8 (Fig D), PSMB9 (Fig E), TAP1 (Fig F), and TAPBP (Fig G). Data is presented as log2 fold change over mock (untreated cells). 4 tumors and 3 normal colon samples were macro-dissected and RNA was isolated, followed by q-RT-PCR analysis (Fig H). (TIFF) [file pone.0179501.s001.tiff]

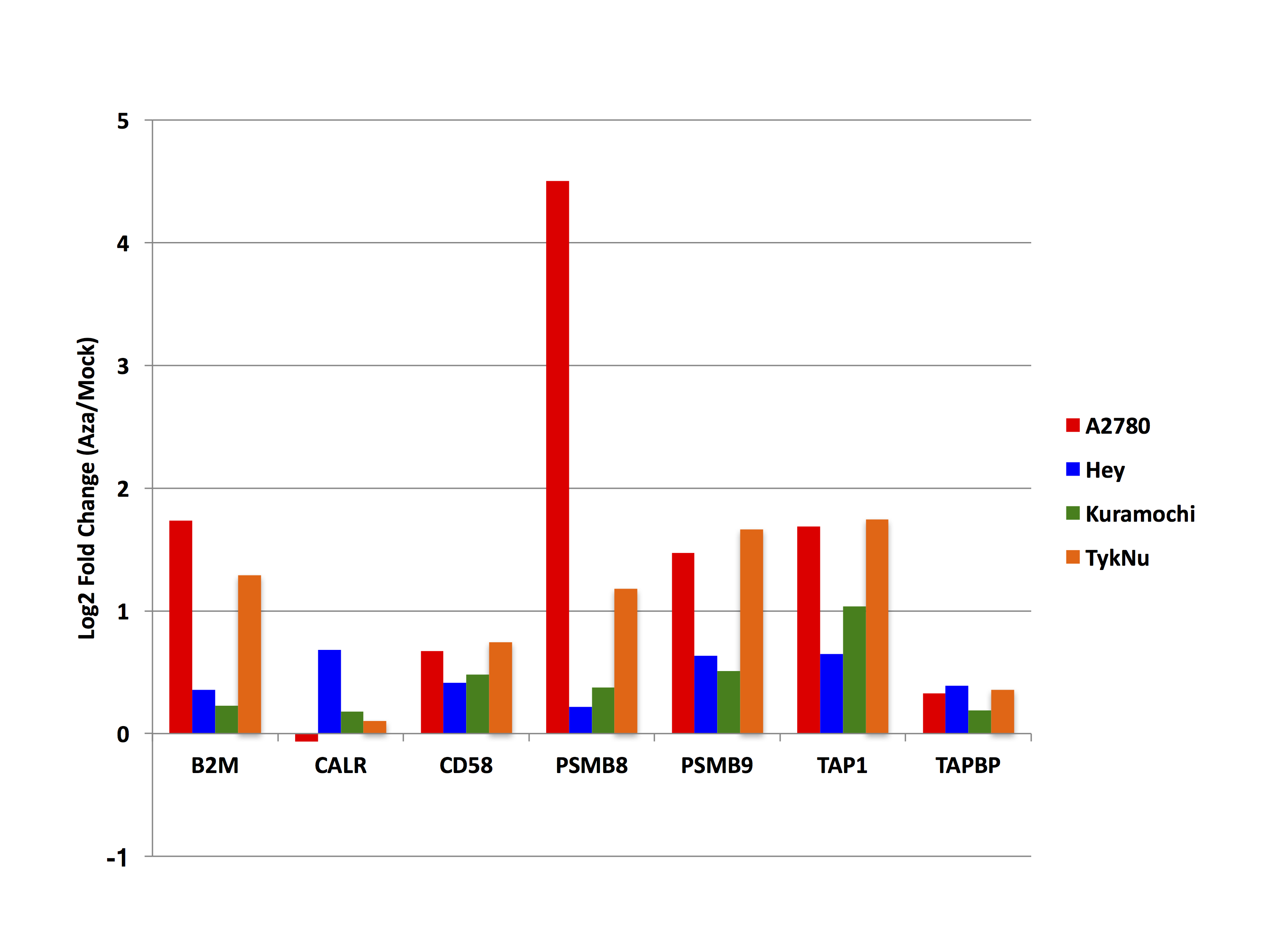

Supplement: S2 Fig — Ovarian cancer cell lines A2780, Hey, Kuramochi, and TykNu were treated with 500 nM of 5-AC every 24 hours for 3 consecutive days and harvested at 10 days after the beginning of treatment. RNA was isolated and made into cDNA. Agilent expression array was performed; data is presented for B2M, CALR, CD58, PSMB8, PSMB9, TAP1, and TAPBP. Data is presented as log2 fold change over mock (untreated cells). (TIFF) [file pone.0179501.s002.tiff]

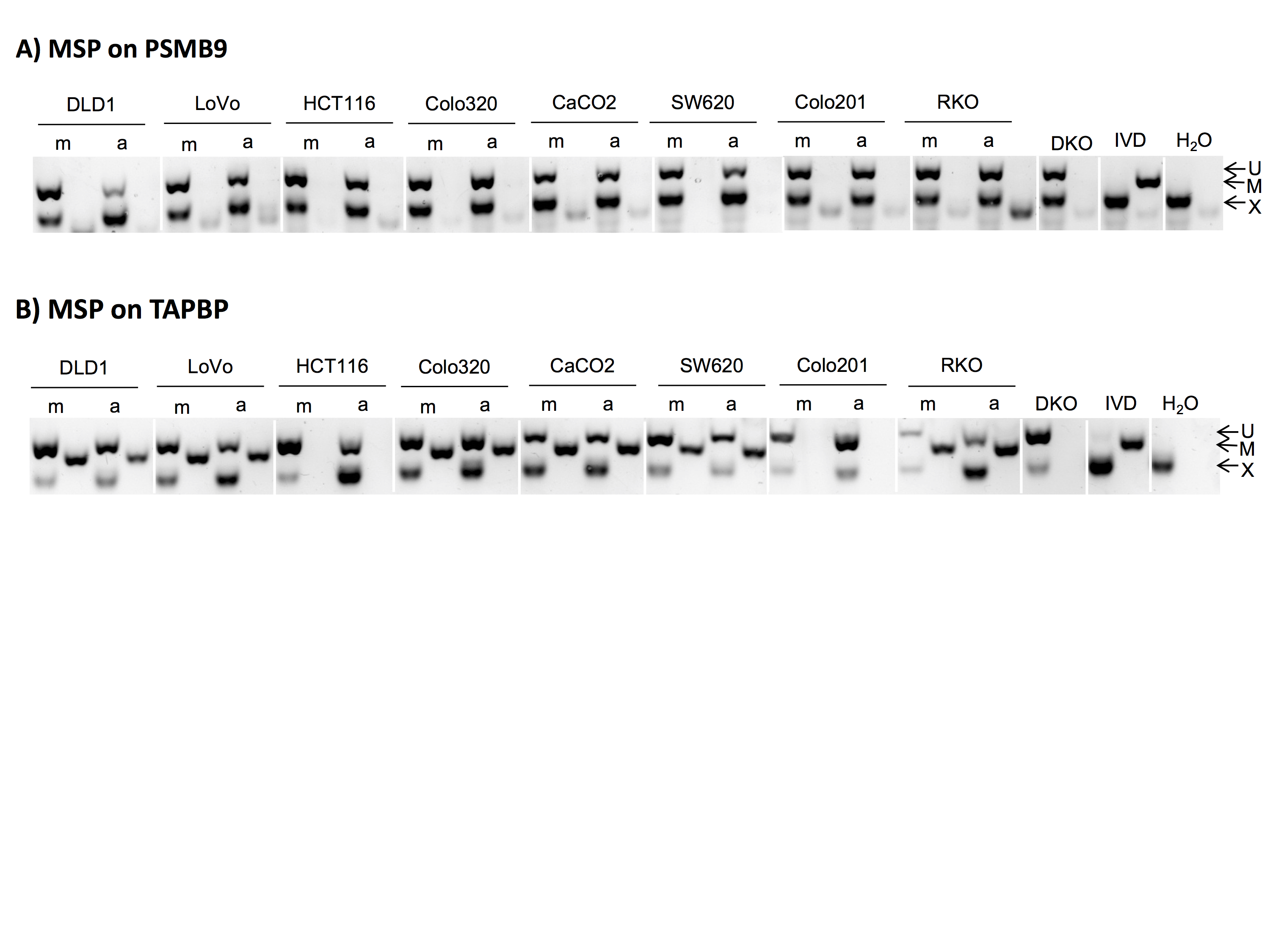

Supplement: S3 Fig — Colon cancer cell lines DLD1, Lovo, HCT116, Colo320, Caco2, SW620, Colo201, and RKO were treated with 500 nM 5-AC for three days, treating every day. DNA was isolated 7 days after beginning treatment and bisulfite treated. Methylation-specific PCR was performed on PSMB9 (Fig A) and TAPBP (Fig B). “m” indicates mock sample, “a” indicates 5-AC treated sample. DKO = unmethylated control, IVD = completely methylated control, H2O = water (no template) control. Arrows to right indicate PCR bands (U = unmethylated, M = unmethylated, X = nonspecific band). (TIFF) [file pone.0179501.s003.tiff]

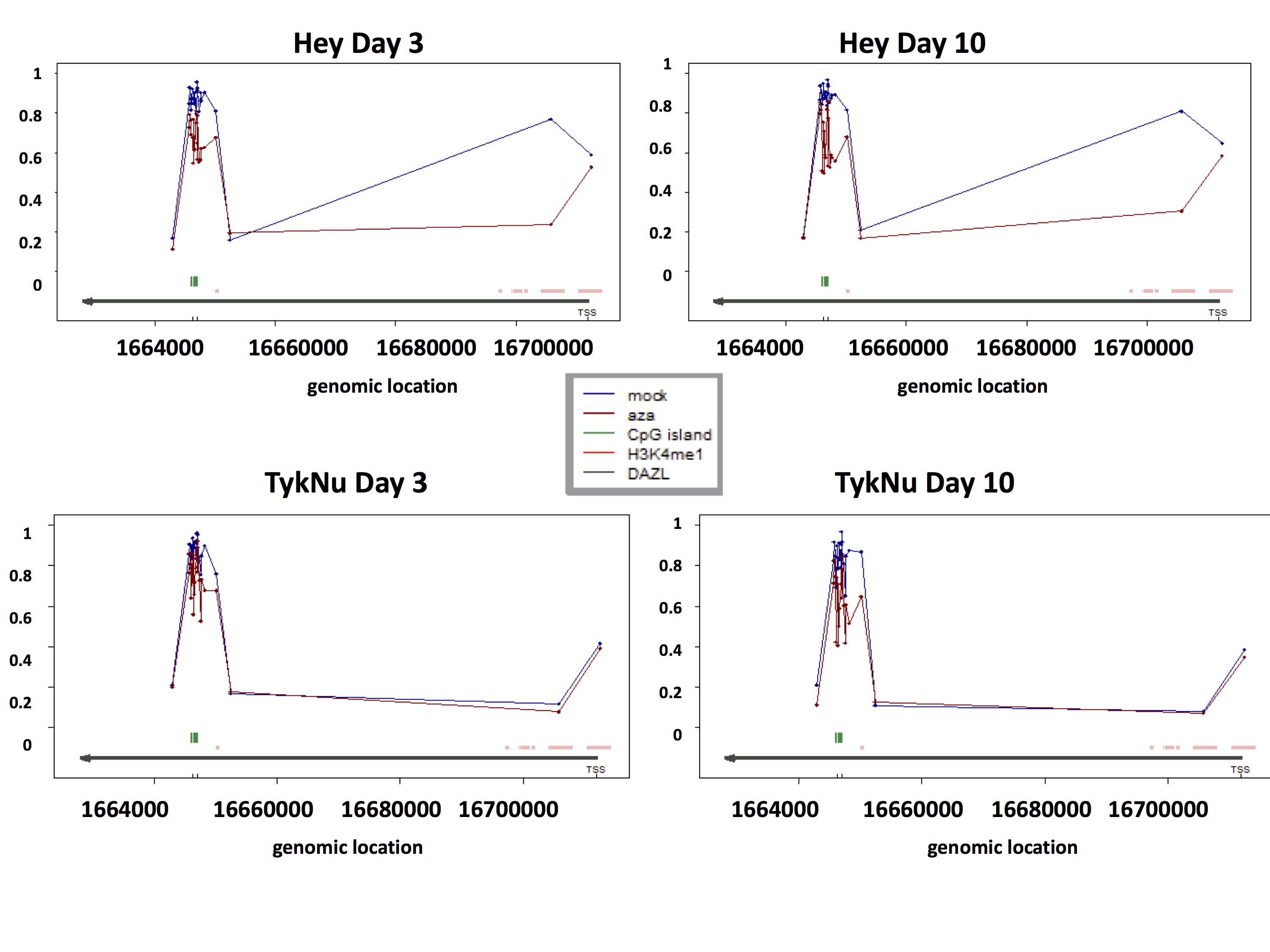

Supplement: S4 Fig — Ovarian cancer cell lines were treated with 500 nM of 5-AC every 24 hours for 3 consecutive days and harvested at 3 and 10 days after the beginning of treatment. DNA was extracted and analyzed using the Infinium 450k methylation array. Results are shown as beta value (percentage methylation) at probes along the promoter region of DAZL (probes shown on x-axis). Blue lines indicate mock samples and red lines indicate 5-AC treated samples. (TIFF) [file pone.0179501.s004.tiff]
